# ADHD-Gaming Disorder Comorbidity in Children and Adolescents: A Narrative Review

**DOI:** 10.3390/children9101528

**Published:** 2022-10-06

**Authors:** Luana Salerno, Leonardo Becheri, Stefano Pallanti

**Affiliations:** 1 INS, Istituto di Neuroscienze, 50121 Florence, Italy; 2 Department of Psychiatry, Albert Einstein College of Medicine, Bronx, NY 10461, USA

**Keywords:** attention-deficit hyperactivity disorder (ADHD), video game, gaming, addiction, children, adolescent, neurobiology, reward

## Abstract

Attention Deficit/Hyperactivity Disorder (ADHD) is a neurobiological condition characterized by developmentally inadequate levels of inattention, hyperactivity, and impulsivity, and a neurobiological disruption in brain neurotransmitters and circuitry causing abnormal responses to rewards. Playing electronic games generates a biological response that activates the neuronal circuits linked to pleasure and reward, and there is a growing attention to this type of activity, which can also turn into a mental health condition. The existence and the boundaries between the functional and the dysfunctional are still a source of debate, with the recognition of ‘Internet Gaming Disorder’ (IGD) as a condition belonging to the broader area of addiction requiring more in-depth study with respect to the DSM-5, while ‘Gaming Disorder’ (GD) was officially recognized as a new diagnosis by the World Health Organization (WHO) in the updated revision of the International Classification of Diseases (ICD-11). Notwithstanding, the suggested criteria for the diagnosis of Gaming Disorder are still debated. Since ADHD has been reported as a risk factor for developing addictions, this narrative review aims to provide the current state-of-the art of the knowledge about the comorbidity between ADHD and Gaming Disorder. For this aim, a literature search was conducted using a combination of specific keywords and the results are discussed within the R-Do-C framework and dimensions, and implications for treatment are considered.

## 1. Introduction

Attention Deficit/Hyperactivity Disorder (ADHD) is a neurobiological condition characterized by developmentally inadequate levels of inattention, hyperactivity, and impulsivity, causing interference with functions in several key domains in life. Epidemiological studies indicate a worldwide prevalence of ADHD in children and adolescents of 5–10% [1] and of 2.8% in the adult population [2]. There is accumulating evidence supporting the notion that ADHD runs in families, as family, adoption, and twin studies showed heritability estimates of 74–80%, for both males and females and for symptoms of inattention and hyperactivity–impulsivity [3,4,5].

When not adequately treated, ADHD can exert significant personal and socioeconomic burdens, determining a very deleterious impact on an individual’s functioning at home, school, in social contexts, and job settings, and causing high costs on society [6]. Adolescents with ADHD are more at risk of being rejected at school, not graduating high school or college, and encountering problems in relationships with peers [7]. Moreover, they show a greater vulnerability to risk-taking behaviors than typically developing peers, including substance use disorders [8], reckless driving, and risky sexual behaviors [6,9,10]. There are several behavior scales, checklists, and clinical interviews that can aid clinicians in the ADHD evaluation process, and they are generally based on DSM-5 or ICD-11 criteria [11,12], which are aligned to enable the diagnosis of three different ADHD presentations: predominantly inattentive, predominantly hyperactive-impulsive, and combined presentations. 

After the inclusion of Internet Gaming Disorder (IGD) in DSM-5 [11] as a condition requiring further study, Gaming disorder (GD) has been recently included in the updated version of the International Classification of Diseases (ICD-11; https://icd.who.int/en (accessed on 3 July 2022)) [12]. In the DSM-5, IGD was defined by a pattern of gaming resulting in significant impairment or distress that was required to meet at least five out of nine criteria, namely, preoccupation, withdrawal, tolerance, loss of control, reduced non-gaming interests, gaming in spite of harms, deception about gaming, gaming as a means to escape or to regulate mood, and conflict/interference caused by gaming [11]. Compared to IGD, ICD-11 defines GD with a stricter monothetic approach, requiring the presence of an enduring gaming behavior, a loss of control over gaming, and a functional impairment caused by gaming, represented by lost life opportunities and negative impacts on normal routines, self-care, social relationships, and responsibilities, for a 12-month period. This difference has been at the center of a vast debate and has recently provoked a panel of experts to gather to evaluate its impact on prevalence estimates and on the possible risk of pathologizing a behavior [13]. The existence of this risk was reasonable, since a multi-center cohort study performed on high-risk adolescents in Korea found a prevalence of IGD of 32.4% according to DSM-5 compared to a prevalence of 6.4% according to ICD-11 criteria [14]. Therefore, the prevalence of GD is strictly dependent on which set of diagnostic criteria the clinician refers to.

Due to the widely recognized association between ADHD and substance and behavioral addictions [6], over the past decade, there has been a proliferation of research examining the frequency of excessive internet use or gaming in people with ADHD, highlighting a frequency of their comorbidity ranging from 29% [15] to 83.3% [16].

In this paper, in view of the growing popularity that video-game-based treatments are receiving as a potential rehabilitation tool in ADHD [17,18], we believe that it is appropriate to evaluate the state-of-the-art research on ADHD-GD comorbidity comorbidity in children and adolescents, so that the clinical implications can be considered as well as future indications for research.

## 2. Materials and Methods

**Figure 1:**
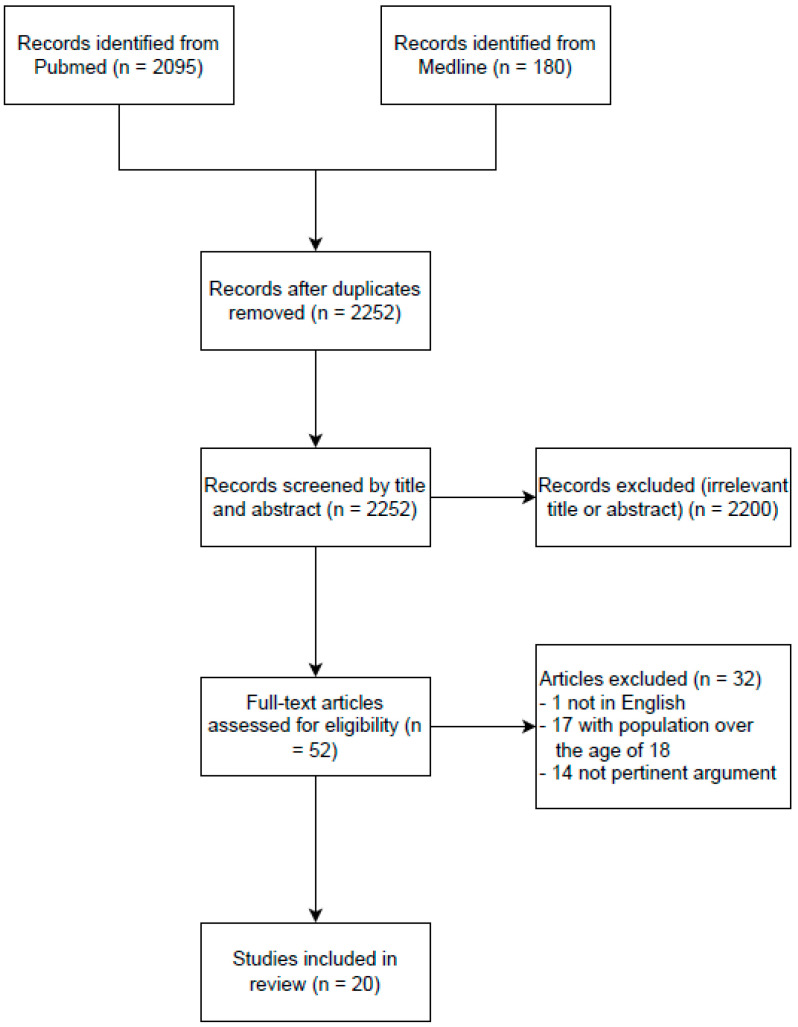
Flow of information through review.

PUBMED and MEDLINE were searched electronically in July 2022 (3–14 July) utilizing a combination of the following keywords and word variants: “*ADHD*” OR “*Attention Deficit Hyperactivity Disorder*” AND “*Gaming disorder*” OR “*Video-game addiction*” OR “*Videogame addiction*” OR “*Gaming addiction*” OR “*Internet gaming disorder*” OR “*Pathological video-game use*” OR “*VGA*” OR “*comorbid psychopathology*”. Firstly, two authors (L.S. and L.B.) independently searched the databases and reviewed titles and abstracts. Thereafter, the two researchers screened full texts to define the eligibility of the study according to the inclusion criteria, i.e., original papers that investigated the ADHD–GD comorbidity with a sample population with an age ≤18. Exclusion criteria were papers not written in English, dissertations, book reviews, and studies that examined that comorbidity in only adults with ADHD. References cited in the selected articles were reviewed and included if considered relevant. The articles referenced for the purpose of this narrative review were published between 2012 and 2022. In case of disagreement during the process, consensus was attained by discussion. The process of eliminating non-relevant papers can be seen in the following flowchart (see Figure 1). 

## 3. Results 

Only 20 studies were found that explored the comorbidity between ADHD and GD and responded to the inclusion criteria. 

### 3.1. Assessment of GD in ADHD 

As visible in Table 1, the optimal diagnostic assessment of GD is still in progress and there is a non-unanimous modality, as different studies have used different rating scales or interviews for assessing its prevalence in children and adolescents with ADHD. Among these, four studies used the Young’s Internet Addiction Scale (YIAS) [19,20,21,22], three used the Chen Internet Addiction Scale (CIA) [23,24,25], two the Internet Addiction Test (IAT) [26,27], one used the Internet Gaming Disorder Interview (IGDI) [28], one the Gaming Addiction Identification Test (GAIT) [29], one the Korean Young’s Internet Addiction Rating Scale (YIAS-K) [30], one the ADITEC questionnaire [31], one the Internet-Gaming Disorder Scale-Short Form (IGDS9-SF) [27], one the Game Addiction Scale for Adolescents (GASA) [32], one the Problem Video Game-Playing Test (PVGT) [33], and one the Computer and Video Game Addiction Scale [34]. Finally, one did not use any rating scales but only the criteria proposed by the DSM-5 [15], and two used reports by parents, teachers, or participants regarding the time spent gaming [35,36]. Gao and colleagues’ meta-analysis included studies where the diagnosis of GD was made by using the DSM-5 criteria or the YIAS or CIAS.

### 3.2. What We Currently Know about The ADHD–GD Comorbidity

Children and adolescents with ADHD and GD have been described to be more dysfunctional video gamers, spending more time engaging in online chatting and gaming daily and during the weekends [24,36]. A different sleep pattern has also been reported, as children and adolescents with ADHD who spent more time online tended to go to sleep later at night, between 2 and 4 a.m., compared to pupils without ADHD who went to sleep between 8:30 and 10:30 p.m. [26]. However, the characteristics of the sleep pattern represent a dimension that has not been investigated by means of specific evaluation tools. 

Regarding the ADHD–IGD/GD relationship, several authors reported ADHD as a risk factor for developing Internet Addiction Disorder (IAD) and IGD [27,31,32], with symptoms of ADHD having ORs of 2.43 (95% CI 1.44–4.11) to present a problematic gaming behavior [29]. Stenseng and colleagues [35] found that while ADHD predicted more time gaming, gaming behavior did not predict more ADHD symptoms, and that time spent gaming did not determine more psychiatric problems at ages 6–10. Instead, Nikkelen and colleagues [38] found that gaming may lead to more ADHD symptoms in early childhood [38] and Gentile et al. [36] described a bidirectional relationship between the time spent gaming and self-reported ADHD symptomatology in Singaporean adolescents. Moreover, they found that exposure to video game violence had a unique effect on attention problems and impulsiveness [36].

Regarding the ADHD core symptom dimensions that were most associated with GD, some authors reported a key role of impulsivity [34,38] and the association of both the combined type and predominantly hyperactive/impulsive ADHD with GD [31], whereas others found more inattentive symptoms in those with both ADHD and GD [24,33], and a trend of a clinically relevant association between the ADHD inattentive type (ADHD/I) and Mobile phone addiction (MPA), especially in females [31]. A male gender was associated with a higher rate of problematic gaming compared to girls [34,35], showing a ratio of more than 5:1, but girls showed greater overall rates of psychiatric symptoms compared to boys [29]. 

Children and adolescents with ADHD and IGD/GD were characterized by poor interpersonal relationships [24,25], more withdrawal tendencies, and a greater loss of control than those without GD and with more emotional problems and disruptive mood dysregulation [23,24]. GD appeared to play a mediating role in raising the risk of disruptive mood dysregulation in children and adolescents with ADHD [25]. Males were exposed to more severe consequences than females, and the negative consequences of excessive gaming were more prevalent in the social domain for males and in the emotional domain for girls [32], who appeared to be characterized by a more unstable mood and with more symptoms of depression [29]. 

Subjects with both ADHD and GD appeared as a heterogeneous group, with some characterized by higher internalizing problems, such as anxiety/depression, withdrawal, and socialization problems at CBCL, and others by externalizing problems and aggressive and rule-breaking behaviors [27]. Therefore, the existence of two different profiles has been hypothesized, and the two emergent profiles (“escape from reality” versus “sensation seeking”) might contradistinguish diverse phenotypes of ADHD plus GD patients. Therefore, the first behavior could be interpreted as an expression of a search for pleasure in “losing control” from reality (a cardinal fingerprint of substance use disorder), while the latter as the search for exciting stimuli. Those with ADHD+GD and internalizing problems would use Internet addiction as a way to escape from daily frustrations derived from poor self-esteem and social anxiety [27], while those with externalizing problems would be characterized by the impulsive need for rapid satisfaction. 

### 3.3. The Impact of ADHD on Gaming Disorder across the Lifespan

The presence of comorbid ADHD in GD patients has been found to be associated with a poor clinical course of GD [20]. Therefore, it appears to be a pathological complication rather than as a functional coping strategy. In a 3-year clinical cohort study, although GD symptoms ameliorated over time despite comorbid ADHD, ADHD symptoms were positively associated with GD symptoms at the baseline, and changes in ADHD symptomatology were greatly associated with those in GD symptoms [20]. The patients with ADHD+GD showed a lower likelihood of recovery than the group with only GD (60% versus 93%, *p* < 0.001) and significantly higher odds of relapse within 1-year (odds ratio, 4.98). Notably, the family environment scores had the greatest impact on GD symptomatology among the clinical covariates and better family conditions were negatively associated with GD symptoms over time. Participants who received psychiatric intervention during the past year had a significantly decreased severity of GD symptoms, leading the authors to state that the assessment and treatment of ADHD and interventions in a family context in children and adolescents with GD may be key factors for improving GD prognosis [20]. The impact of pharmacological ADHD treatments on GD has been investigated by Chang and colleagues [23], who reported that the effectiveness of the treatment of GD in the presence of comorbid ADHD would be satisfactory if underlying symptoms of inattention, hyperactivity/impulsivity, and oppositional defiant disorder were under control by ADHD pharmacotherapy [23]. Indeed, in youth with internet addiction and ADHD, also presenting symptoms of DMDD, methylphenidate, atomoxetine, and the combination of methylphenidate or atomoxetine with aripiprazole should be used as good drug choices for the treatment of GD [23].

### 3.4. Brain Structural and Functional Connectivity in ADHD and GD

A comparative meta-analysis of whole-brain voxel-based morphometry (VBM) studies found disorder-specific grey-matter volume (GMV) abnormalities in the putamen in patients with IGD and in the orbitofrontal cortex in those with ADHD, while both conditions shared a lower GMV in the prefrontal cortex [37]. Functional magnetic resonance-imaging (fMRI) research comparing IGD versus ADHD found that ADHD was associated with a disorder-specific hypoactivation of the left median cingulate cortex (MCC), middle temporal gyrus (MTG), right caudate nucleus, and left middle frontal gyrus [37]. Alternatively, individuals with IGD were characterized by the activation of the bilateral precuneus/cingulate cortex (CC), right OFC, MTG, left precentral gyrus, bilateral IFG, and right caudate nucleus [37]. Therefore, the structural and functional alterations in both ADHD and IGD subjects were in the PFC areas, and, more specifically, in those associated with reward, such as the anterior cingulate cortex (ACC).

Another study that investigated whether ADHD and IGD share a similar degree of brain functional connectivity (FC) between the frontal and subcortices [30] found that, at baseline, both subjects with ADHD and those with IGD were characterized by reduced FC from the right-middle frontal gyrus to the caudate and from the left cingulate to the caudate, compared to matched healthy controls (HC). After the baseline assessment, the patients received treatment including CBT and medication for ADHD (methylphenidate or atomoxetine) and IGD (i.e., bupropion or escitalopram), and after one year of treatment the authors found enhanced cortical brain activity within the right-middle frontal gyrus as well as the FC between the cortex and subcortex in those with a good prognosis compared to those with a poor prognosis (i.e., those adolescents who after 1 year of treatment did not reduce their CGI-I ADHD scores of 1 or 2).

A quantitative electroencephalography (qEEG) study performed by Park and colleagues [19] found lower relative delta band power and greater relative beta band power values in temporal areas in an ADHD+IGD group in comparison with patients with only ADHD, while the relative theta power in frontal areas was significantly higher in the subjects with only ADHD in comparison with the healthy controls (HC). The ADHD+IGD group showed no theta band inter-hemispheric coherence diversity in the frontal and central regions and relative beta power compared to the HC group. Such findings led the authors to hypothesize that a higher susceptibility to attention problems may be associated with internet game play as a means to enhance attentional capacity, which is in turn reflected by the beta power in the ADHD+IGD group, which was similar to that found in HC. Moreover, the continual visuospatial working memory and executive function activation during internet gaming may determine an increase in neuronal connectivity among the fronto-central, parieto-occipital, and temporal areas, which is reflected by an increase in inter-hemispheric coherence in those regions. In addition, Han and colleagues (2017) [22] found a hyper-connectivity between those regions related to visual–auditory multi-tasking, motion detection, and the efficient processing of dynamical audiovisual stimuli, leading the authors to hypothesize a training consequence of extended game play.

## 4. Discussion 

The main goal of this review was to examine the current state of the literature regarding the ADHD–GD comorbidity in order to inform clinicians about potential underpinnings that may explain their frequent co-occurrence and implications for treatment. Regarding the clinical features of such a co-occurrence, our review confirmed that multiple ADHD symptoms may be risk factors for problematic gaming, but not all studies confirmed the presence of a bidirectional relationship between the two conditions. As previously reported, males with ADHD show a high risk of developing pathological gaming behaviors [26,39,40,41]; however, gender issues in ADHD should be cautiously considered, as boys are generally overrepresented among those presenting gaming disorder [40,41], ADHD is more frequently diagnosed in males than in females at developmental ages [42], and potential different patterns of addiction characterizing males and females with ADHD are emerging (Mobile phone addiction or MPA versus GD) [31]. 

The importance of shedding light on the reasons for such a frequent comorbidity is due to the data indicating that having ADHD is associated with a more persistent course of IGD, decreased recovery rates, and higher rates of recurrence [20,43], and those supporting that the effectiveness of GD treatment depend on how well the ADHD symptoms and their severity are being controlled [20,23,44,45]. This is possibly due to a shared mechanism of reward and sensitization primarily mediated by dopamine, which found support by the efficacy of stimulant medication in reducing both ADHD and IGD symptoms’ severity [23].

Notwithstanding the negative consequences of gaming in youth with ADHD, there is increasing evidence supporting the notion that video games may enhance cognitive performance thanks to continuous feedback [46], thereby increasing attention [47], inhibitory control [48], and increasing arousal by promoting enhanced motivational performance [48]. In accordance, video games have been proposed as a therapeutic tool in ADHD [17,49,50,51,52,53], with some research providing support to the clinical application of Serious Games in the rehabilitation training of ADHD [54,55,56,57,58], while others provide less support [59,60,61].

Currently, it is not clear why some ADHD individuals are more susceptible to an excessive and pathological gaming behavior than those without ADHD, and whether there is a subgroup that could take advantage of video games for rehabilitation. We believe that in order to clarify this important issue, clinical assessments of the related disability, and not just quantitative assessments, as well as insights into their underlying neural disturbances could help.

In this regard, in the ADHD–GD relationship, it is possible to hypothesize a disorder of connectivity in some areas of the brain that largely overlap, while others seem to have their own specificity. Research on common structural and functional aberrations in ADHD and GD found that both GD and ADHD are characterized by deficits in the reward circuitry, including the anterior cingulate cortex (ACC), the prefrontal and orbitofrontal cortex (PFC and OFC), the striatum (including the caudate nucleus, putamen, and globus pallidus), the amygdaloid nucleus, and the thalamus [62,63,64]. A key region seems to be the anterior cingulate cortex (ACC), which is known to modulate the neural activity of the default-mode network and executive control network [65] and participates in processes including cognitive control [66], the regulation of emotions [67], and reward-relative decision making [68]. A significantly smaller ACC gray matter volume in ADHD and a decreased ACC volume have been found to be directly associated with attentional deficits [69], and individuals with IGD showed problems in controlling their compulsion for gaming, notwithstanding the negative consequences because of the impaired cognitive control of ACC [70]. Concerning the differences among the two disorders, a disorder-specific activation in the precuneus has been found among GD subjects [37], which is the area related to memory retrieval, visual imagery, and attention [71], and it has also been found that a great activation in the precuneus may underly the processing of gaming cues, contributing to the cue-induced craving for online gaming [37]. ADHD subjects showed a particular activation in the fusiform gyrus, which the through connection between the striate cortex and the inferior temporal lobe plays a key role in high-level visual/cognitive functions [72]. Moreover, by mediating various stimuli it could make it difficult for ADHD individuals to concentrate on a given task [37]. 

Using a Research Domain Criteria (R-Do-C) framework, ADHD deficits appear to fit the domains of cognitive systems, because of the impairment of attention [73], working memory [74], and of the timing-related functions (motor and perceptual timing and temporal foresight) [75]; the positive valence system, with the sub-constructs of reward anticipation, receipt, and delay [76,77,78,79,80]; the domain of social processes, because of altered facial emotion processing [81,82] due to the frontostriatal dysfunction [83]; the arousal and regulatory systems because of the frequently disordered sleep patterns [84,85]; and the sensorimotor systems, because of the impairments in motor control underlying the excessive moving or talking characterizing individuals with ADHD [84,85]. People with substance use disorder showed impairments in the domains of the negative and positive valence systems, cognitive systems, systems for social processes, and arousal and regulatory systems [86]. It has been proposed that the disordered brain function in ADHD and GD overlaps with the executive function, incentive salience, and negative emotionality domains [87], and that deficits in the cognitive system and negative emotionality interactively increase ADHD and GD symptomatology [23]. This would occur as children and adolescents with ADHD tend to avoid complicated tasks due to impairment in executive functions, and that because inattentive ADHD symptoms they tend to overplay, so that the internet gaming determines a sort of disruption of the incentive salience domain [87]. Consequently, ADHD children and adolescents firstly develop the symptoms of distorted liking or wanting gaming firstly, followed by the withdrawal-negative affect when they are stopped from playing games [23]. In this process, GD constitutes an aggravating factor for ADHD symptoms, determining an increase in ADHD symptoms’ severity by leading children and adolescents to experience more symptoms of irritability, loss of control, and craving/withdrawal due to addiction to gaming [88].

It is also true that recent reviews have reported how video games and playing serious games can improve several areas that are impaired in ADHD, such as attention, executive functions, hyperactivity and impulsivity, emotional regulation, and motor and visual skills [17,18,89], as well as modifying EEG activity in ADHD+IGD adolescents in comparison with those with ADHD only [19] in relation to the relative beta and theta power. As methylphenidate was shown to increase beta power in centro-parietal regions, with improvements in behavioral and cognitive symptoms [90], it is possible that gaming by stimulating synaptic dopaminergic neurotransmission can determine an attentional capacity enhancement similar to that produced by methylphenidate [91]. Moreover, the continuous gaming induced complex competitions and interactions between inter-hemispheric neurons making ADHD+GD individuals not different by healthy controls [19]. Moreover, ADHD is generally associated with abnormal Default Mode Network activity [92] and it has recently been found that DMN hubs deactivated in learning conditions become more like a game, with higher self-reported engagement as well as higher learning scores in the game-based condition [93]. 

How can we use the collected information to understand the pros and cons of using video games in ADHD rehabilitation, considering the vulnerability of developing addictive behaviors with respect to video games and the positive association between ADHD symptoms severity and excessive gaming? Our review confirmed the existence of several factors implicated in the development of GD in ADHD. Children and adolescents with the disorder can use gaming for overcoming their impairment in social functioning and a sense of loneliness, and, therefore, as a coping modality to escape problems of daily life [94], negative and depressed moods [40], and even boredom interwoven with inattention [95,96]. In this way, gaming can provide a socially safe place free of demands and obligations [97] and pleasurable states of flow or escape related with, among others, diminished negative effects [35]. Furthermore, as gaming is a particularly satisfying engagement for children and adolescents with ADHD, it constitutes a quick satisfaction of the impulsive need for immediate gratification, which is constantly nourished by the particular nature of the game and the possible scenarios involved, and it can provide a sort of control that is reached through the mastery of the game’s challenges [35]. In this regard, excessive gaming may be sustained by stimulatory effects on reward and sensitization [98], in the same way that long-term modifications in the brain reward circuit can sustain substance dependence [26]. Therefore, is has been suggested that video games could play a real role in ADHD rehabilitation if the visual effects of the game design are not too eye catching, the rules and gameplay of the game focus on cultivating concentration and suppressing impulses, and the game characters and scenes correspond to real life [89]. The family environment should be also considered because of its influence in the emergence of problematic gaming behavior [99,100] and the persistence of GD symptoms over time [20]. Therefore, even when video games are considered for ADHD treatment, parent training and individual psychological interventions should always be provided within the framework of a multimodal approach and considering the effectiveness of specialized psychological programs for patients with GD [101,102,103] and the parent training for ADHD [104] even when performed online [105]. Finally, since a protracted exposure to addictive agents has been shown to result in disruptions in several circuits responsible for maintaining addiction due to neural adaptations, neurocircuit-based interventions could also be taken into account, thanks to their potential to target the neuroanatomical structures that work as ‘nodes’ within these circuits [106], with protocols specifically created for behavioral addictions [107]. 

It is important to point out that the collected findings should be considered in light of several limitations. Although our data provide confirmation of the link between ADHD and GD, since this was a narrative review, we did not test the strength of such an association and did not assess the quality of the studies included or the data’s extraction. The same frequency of co-occurrence of these conditions may be rather variable depending on the different instruments and criteria used for the diagnosis. Indeed, the studies vary with respect to the tools that have been used, with some carried out before the definition of the criteria of GD/IGD in the DSM-5 and ICD-11, and others using measures of internet addiction developed through an adaptation of gambling disorder or more general addiction criteria. Therefore, not all these tools can be considered suitable for the diagnosis of GD, confirming the criticalities reported regarding the diagnostic criteria and the danger of overdiagnosis [13,108,109,110]. In addition, there is a lack of replication studies and of blinded assessors, with many studies being characterized by small sample sizes and a low degree of systematization of the research [17,18]. Finally, we included only research published in English, and many studies had male-dominated population samples; consequently, generalizations to female individuals should be made with caution. Therefore, more well-designed RCTs, with larger sample sizes balanced by sex and a better methodology, are necessary in order to better understand the common and distinct underpinnings of ADHD and GD. 

## 5. Conclusions

Collectively, our findings led us to state that although there is an increasing number of publications on the subject, there are several aspects that future research should address to better define the common and specific underpinnings of the ADHD+GD comorbidity that can inform treatments and promote prevention. A shared definition of gaming disorder and the development of reliable diagnostic tools with specific cut-offs are necessary to avoid the risk of its overdiagnosis and the failure to clearly identify the dysfunctional neural mechanisms underlying its frequent co-occurrence with ADHD, as well as the eventual recognition of new subtypes of the ADHD plus GD condition. Indeed, treatments will be necessarily different depending on the dysfunctions in the positive valence or negative valence systems (i.e., the need of immediate reward versus escape from unpleasant situations) that will be found in the already heterogeneous ADHD population. Only with a clear identification of all the psychopathological dimensions involved and the underlying neural mechanisms will it be possible to enhance the translation of clinical research into practice, and develop the best treatment for a given individual, including the emerging non-invasive neuromodulation techniques, in line with a personalized approach. 

## Figures and Tables

**Table 1:** The table displays study characteristics, design, and outcome of the studies that were found relevant to the aim of our review.

Authors (Year)	Study Design	Sample	Age (Years)	Assessment Tool for IGD	Outcomes
Hygen, B. W. et al. (2020) [28]	Cross-sectional	*N* = 702 children and adolescents	10–14	Internet-Gaming Disorder Interview (IGDI); Child and Adolescent Psychiatric Assessment (CAPA)	At all ages there was a positive and significant correlation between IGD symptoms and those of ADHD, anxiety, depression, and ODD/CD. More IGD symptoms were associated with more ADHD, anxiety, and ODD/CD symptoms but not depression at the between-group level.
Vadlin, S. et al. (2016) [29]	Cross-sectional	Community sample: *N* = 1868; Clinical sample: *N* = 242 adolescents (male = 834; 44.6%; female = 1034; 55.4%)	Community sample: aged 12–16 (mean age = 13.9); Clinical sample: aged 12–18 (mean age = 15.39)	Gaming Addiction Identification Test (GAIT); for psychiatric symptoms: Adult ADHD Self-Report Scale Adolescent version (ASRS-A); Depression Self-Rating Scale Adolescent version (DSRS-A); Spence Children’s Anxiety Scale (SCAS); psychotic-like experiences (PLEs)	Girls constituted 55.4% of the community sample and 69.8% of the total sample (*p* < 0.001). A total of 21.3% of the problematic gamers belonging to the community sample had symptoms of ADHD vs 42.3% belonging to the clinical sample (*p* = 0.019). ADHD symptoms were associated with ORs of 2.43 (95% CI, 1.44–4.11) with respect to coexisting problematic gaming.
Han, D. H. et al. (2021) [30]	Case-control	*N* = 113 adolescents (29 ADHD + IGD; 20 pure IGD; 26 pure ADHD; 38 HC)	14–15	Korean–Wechsler Adult Intelligence Scale (K-WAIS); Korean version of Dupaul’s ADHD-Rating Scale (K-ADHD); Korean Young’s Internet Addiction Rating Scale (YIAS-K); Children’s Depression Inventory (CDI); Beck Anxiety Inventory (BAI); Behavioral Inhibitory System/Behavioral Activation System (BIS/BAS) Scale; Clinical Global Impression-Improvement Scale (CGI-I)	At the baseline, in the all-ADHD and all-IGD groups, the Functional Connectivity (FC) from the right-middle frontal gyrus to the caudate as well as the FC from the left cingulate to caudate were reduced in comparison to HC. After one year of treatment for ADHD and IGD symptoms there was an increase in the FC between the cortex and subcortex in both all-ADHD and all-IGD participants with a good prognosis in comparison with participants with poor prognoses.
Park, J. H. et al. (2017) [19]	Case-control	ADHD+IGD group: *N* = 16 male adolescents; ADHD-only group: *N* = 15 male adolescents; HC group: *N* = 14 male adolescents.	ADHD+IGD group: mean age = 14.6 ± 1.9; ADHD-only group: mean age = 13.7 ± 0.8; HC group: mean age = 14.4 ± 1.7	Korean ADHD-Rating Scale (K-ARS); Young’s Internet Addiction Scale (YIAS)	Adolescents with ADHD who also had a problematic Internet use showed differences in qEEG in comparison with those with only ADHD; specifically, ADHD+IGD comorbidity presented lower relative delta power and greater relative beta power compared to adolescents with only ADHD.
Chang, C. H., et al. (2020) [23]	Cross-sectional	*N* = 101 ADHD children and adolescents (49 without Internet Addiction; 52 with Internet Addiction. Male = 69, 68.32%; Female = 32, 31.68%)	7–18	Chen Internet Addiction Scale (CIA); Swanson, Nolan and Pelham, Version IV Questionnaire (SNAP-IV)	Internet-addicted ADHD youths showed increased comorbidity with DMDD, such as emotional dysregulation, than ADHD youths without IGD and had poorer interpersonal relationships. The efficacy of IGD treatment was good when the underlying ADHD symptomatology was controlled. IGD can lead to severe emotional dysregulation.
Menéndez-García, A. et al. (2022) [31]	Case-control	*N* = 112 children and adolescents (51 with ADHD; 61 without ADHD)	7–17	TEA questionnaire for the assessment of executive function and ADHD (ATENTO); ADITEC questionnaire (ADITEC)	ADHD is a risk factor for developing IAD and IGD, with good social adaptation buffering such an association. ADHD combined type and predominantly hyper active/impulsive type were each associated with IGD. Gender vulnerability to IGD and MPA was reported since there was an association between female gender and MPA and an association between male gender and IGD.
Lee, J. et al. (2021) [20]	Prospective cohort	*N* = 255 patients (128 = Pure-IGD, 127 = ADHD-IGD. Male = 246, 96.47%; Female = 9, 3.53%)	11–42	Young’s Internet Addiction Scale (YIAS); Korean ADHD-rating scale (ADHD-RS); Beck’s Depression Inventory (BDI); Beck’s Anxiety Inventory (BAI); Social Avoidance and Distress Scale (SADS); Family Environment Scale (FES)	Individuals with both ADHD and IGD showed a lower recovery probability compared to individuals with only (pure) IGD (*p* < 0.001) and a higher probability of recurrence over the follow-up period (*p* = 0.006). Changes in symptoms of ADHD were greatly associated with changes in symptoms of IGD.
Haghbin, M. et al. (2013) [34]	Cross-sectional	*N* = 326 high school students. 146 (49.1%) females, 166 (50.9%) males	14–18	Computer and Video Game Addiction Scale; Self-control Scale; Diagnostic Checklist and Self-report Scale; Grade Point Average (GPA)	There was a significantly different relationship between self-control, video game addiction, and academic achievement between normal and ADHD students. The shared factor between ADHD, self-control, and video game addiction was impulsivity. Males showed higher probability of being addicted to video games than females.
Chang, Y. C. et al. (2021) [24]	Cross-sectional	*N* = 102 children and adolescents (Male = 70, 68.63%; Female = 32, 31.37%)	7–18 (mean age = 11.16 ± 3.35)	Chen Internet Addiction Scale (CIA); Swanson, Nolan, and Pelham, Version IV Questionnaire (SNAP-IV)	Youth with ADHD showed more pathological videogaming activity, greater loss of control, and more conflictual and withdrawal tendencies compared to youth with ADHD but without IGD. In those with IGD, there was a more severe inattentive symptomatology, more emotional difficulties, and more mood dysregulation disorders. Moreover, they spent more time in gaming or chatting online daily and during weekends, and their interpersonal relationships were poorer than those without IGD.
Kim, M. et al. (2020) [21]	Case-control	*N* = 46 male IGD+ADHD patients; *N* = 48 male IGD-ADHD patients; *N* = 34 HC	15–26	Young’s Internet Addiction Scale (YIAS), Korean Kiddie Schedule for Affective Disorders and Schizophrenia; Dupaul’s ADHD scale—Korean version (K-ARS-P)	Symptomatic associations between IGD and ADHD were found and abnormal increases in some structural connections within circuitry pertaining to inhibitory function or sensory integration can point out how the ADHD comorbidity is associated with addiction severity in IGD.
Weinstein, A. et al. (2015) [26]	Case-control	*N* = 100 schoolchildren and adolescents (*N* = 50 with ADHD; *N* = 50 without ADHD)	13–15	Young’s Internet Addiction Test (IAT)	Children and adolescents with ADHD were more addicted to the internet, spent more time online, and went to sleep later at night compared to those without ADHD.
Stenseng, F. et al. (2020) [35]	Prospective cohort	*N* = 905 children at T1 (Male = 458, 50.6%; Female = 447, 49.4%); *N* = 752 children at T2 (Male = 380, 50.5%; Female = 372, 49.5%); *N* = 661 children at T3 (Male = 322; 48.7%; Female = 339, 51.3%)	6–10	Preschool Age Psychiatric Assessment/Child and Adolescents Psychiatric Assessment (PAPA/CAPA); parents’ reports on gaming behavior	At age 6, more ADHD symptoms and emotional problems did not predict increased gaming activity at age 8, whereas more ADHD symptoms at age 8 predicted increased gaming activity at age 10. Thus, the time spent gaming did not predict more psychiatric problems at these ages, but children with greater ADHD symptomatology were more likely to increase their amount of gaming during middle childhood.
Han, D. H. et al. (2017) [22]	Case-control	*N* = 151 male children and adolescents (*N* = 78 with IGD, *N* = 73 without IGD)	10–19	Young’s Internet Addiction Scale (YIAS); Beck Depression Inventory (BDI); Beck Anxiety Inventory (BAI); Korean ADHD-rating scales (K-ARS)	Chronic exposure to Internet game play was associated with heightened connectivity between the salience network (anterior insula and dorsal anterior cingulate) and frontal eye fields, between ipsilateral DLPFC and left TPJ, and between motor cortex, auditory cortex, and SMA, which was not explained by comorbidities such as ADHD. Increased connectivity hypothesized as a training effect of prolonged game play, reflecting adaptive functional gain in individuals with a pattern of protracted internet game use.
Berloffa, S. et al. (2022) [27]	Case-control	*N* = 108 children and adolescents (Male = 96, 89%; Female = 12, 11%)	8–18 (mean age = 11.7 ± 2.6)	Internet Addiction Scale (IAT); Internet Gaming Disorder Scale-Short Form (IGDS9-SF); Clinical Global Impression-Severity score (GCI-S); Children Global Assessment Scale (C-GAS); Conners’ Parent Rating Scale - Revised: Short Form (CPRS-R:S); Use, Abuse, and Dependence on Internet (UADI); Wechsler Intelligence Scale for Children—Fourth Edition (WISC-IV)	ADHD+IGD patients showed more severe ADHD symptoms, more withdrawal/depression and socialization problems, and greater salience of addiction and evasion dimensions. The binary logistic regression revealed that the degree of inattention had a greater weight in determining IGD.
Tzang, R. F. et al. (2022) [25]	Case-control	*N* = 102 ADHD children and adolescents (Male = 70, 68.63%; Female = 32, 31.37%	8–15	Chen Internet Addiction Scale (CIAS); Swanson, Nolan, and Pelham Version IC Questionnaire (SNAP-IV)	Youth with ADHD+IGD were significantly more likely to be characterized by poor interpersonal relationships and DMDD-like symptomatology compared to those with only ADHD. IGD played a mediating role in the increase in the risk of disruptive mood dysregulation in youths with ADHD.
André, F. et al. (2022) [32]	Cross-sectional	*N* = 144 children and adolescents (Male = 69, 50.4%; Female = 68, 49.6%)	8–18	Game Addiction Scale for Adolescents (GASA)	ADHD increases the risk of over utilization of computer games, and while for boys the negative consequences had a social direction, they were more emotional for girls.
Mazurek, M. O. et al. (2013) [33]	Cross-sectional	*N* = 141 male children and adolescents (*N* = 56 with ASD, *N* = 44 with ADHD, *N* = 41 TD)	8–18	Problem Video Game-Playing Test (PVGT); Vanderbilt Attention Deficit/Hyperactivity Disorder Parent Rating Scale (VADPRS); Social Communication Questionnaire-Current (SCQ)	Boys with ASD and ADHD showed higher risk for problematic gaming than boys with TD. Inattentive symptomatology was strongly associated with problematic gaming for both groups.
Gao, X.et al. (2021) [37]	Meta-analysis	*N* = 5454 patients with ADHD and/or IGD compared to HC (Male = 4189, 76.81%; Female = 1265, 23.19%)	9–37	IGD diagnosed by using DSM-5 or YIAS or CIAS	IGD and ADHD have common and distinctive structural and functional alterations. IGD showed disorder-differentiating structural alterations in the putamen while ADHD showed aberrations in the orbitofrontal cortex. Disorder-differentiating fMRI activations were predominantly evident in the precuneus among IGD patients and common impairing function connection was present in the reward circuitry.
Nikkelen, S. W. et al. (2014) [38]	Meta-analysis	Children and adolescents with ADHD versus control group (Female 42.27%; Male 57.73%)	0–18 years	Child Behavior Checklist (CBCL); Strenghts and Difficulties Questionnaire (SDQ); Continuous Performance Task (CPT); Attentional Networks Test (ANT); Matching Familiar Figures Test (MFFT); a measure of overall media use	Small significant association between ADHD-related behaviors and use of media. Even though media use was more strongly related to problems with attention than to impulsivity, such a difference was only marginally significant. The ADHD–media use relationship was stronger for boys than for girls.
Gentile, D.A. et al. (2012) [36]	Prospective cohort	*N* = 3034 children/adolescents	8–17 years (mean age = 11.2, *SD* = 2.1)	Current ADHD Symptoms Scale Self-Report; Barratt Impulsiveness Scale-11; Self-reports of gaming behavior	Results suggested a bidirectional relationship between video game-playing and ADHD. Children and adolescents with higher impulsiveness and attention difficulties spent more time playing video games, which in turn intensifies consequent problems in attention and impulsiveness.

ADHD—Attention Deficit and Hyperactivity Disorder; ODD—Oppositional Defiant Disorder; CD—Conduct Disorder; IGD—Internet-Gaming Disorder; qEEG—Quantitative Electroencephalogram; MPA—Mobile phone addiction; DLPFC—dorsolateral prefrontal cortex; TPJ—Temporoparietal Junction; SMA—Supplementary Motor Area; DMDD—Disruptive Mood Dysregulation Disorder; fMRI—Functional magnetic resonance imaging; ASD—Autism Spectrum Disorder; HC—Healthy Controls; TD—Typical Development; *SD*—Standard Deviation.

## Data Availability

The original contributions presented in the study are included in the article, further inquiries can be directed to the corresponding author.
